# Obesity and risk of death or dialysis in younger and older patients on specialized pre-dialysis care

**DOI:** 10.1371/journal.pone.0184007

**Published:** 2017-09-05

**Authors:** Ellen K. Hoogeveen, Kenneth J. Rothman, Pauline W. M. Voskamp, Renée de Mutsert, Nynke Halbesma, Friedo W. Dekker, for the PREPARE-2 Study Group

**Affiliations:** 1 Department of Clinical Epidemiology, Leiden University Medical Center, Leiden, The Netherlands; 2 Department of Nephrology, Jeroen Bosch Hospital, Den Bosch, The Netherlands; 3 RTI Health Solutions, RTI International, Research Triangle Park, North Carolina, United States of America; 4 Usher Institute of Population Health Sciences and Medical Informatics, University of Edinburgh, Edinburgh, United Kingdom; The University of Tokyo, JAPAN

## Abstract

**Background:**

Obesity is associated with increased mortality and accelerated decline in kidney function in the general population. Little is known about the effect of obesity in younger and older pre-dialysis patients. The aim of this study was to assess the extent to which obesity is a risk factor for death or progression to dialysis in younger and older patients on specialized pre-dialysis care.

**Method:**

In a multicenter Dutch cohort study, 492 incident pre-dialysis patients (>18y) were included between 2004–2011 and followed until start of dialysis, death or October 2016. We grouped patients into four categories of baseline body mass index (BMI): <20, 20–24 (reference), 25–29, and ≥30 (obesity) kg/m^2^ and stratified patients into two age categories (<65y or ≥65y).

**Results:**

The study population comprised 212 patients younger than 65 years and 280 patients 65 years and older; crude cumulative risk of dialysis and mortality at the end of follow-up were 66% and 4% for patients <65y and 64% and 14%, respectively, for patients ≥65y. Among the <65y patients, the age-sex standardized combined outcome rate was 2.3 times higher in obese than those with normal BMI, corresponding to an excess rate of 35 events/100 patient-years. After multivariable adjustment the hazard ratios (HR) (95% CI) for the combined endpoint by category of increasing BMI were, for patients <65y, 0.92 (0.41–2.09), 1 (reference), 1.76 (1.16–2.68), and 1.81 (1.17–2.81). For patients ≥65y the BMI-specific HRs were 1.73 (0.97–3.08), 1 (reference), 1.25 (0.91–1.71) and 1.30 (0.79–1.90). In the competing risk analysis, taking dialysis as the event of interest and death as a competing event, the BMI-specific multivariable adjusted subdistribution HRs (95% CI) were, for patients <65y, 0.90 (0.38–2.12), 1 (reference), 1.47 (0.96–2.24) and 1.72 (1.15–2.59). For patients ≥65y the BMI-specific SHRs (95% CI) were 1.68 (0.93–3.02), 1 (reference), 1.50 (1.05–2.14) and 1.80 (1.23–2.65).

**Conclusion:**

We found that obesity in younger pre-dialysis patients and being underweight in older pre-dialysis patients are risk factors for starting dialysis and for death, compared with those with a normal BMI.

## Introduction

The global prevalence of end-stage renal disease (ESRD) that requires renal replacement therapy (RRT) has risen steadily and has more than doubled since 1990 [1,2]. Most notably, the proportion of ESRD patients aged 65 years or older has grown considerably, comprising more than 66% of all maintenance dialysis patients in industrialized countries. The number of ESRD patients aged 65–74 years and >75 years grew from 2005 to 2010 by 11% and 4%, respectively [3]. Identifying potentially modifiable risk factors for ESRD and death is important for targeted prevention.

The prevalence of obesity among adults aged 20 to 74 years has increased in the past 20 years from 15 to 35% in the United States, and from 10% to >30% in the Netherlands [4,5]. Obesity is associated with accelerated decline of kidney function as well as mortality in the general population, as well as among kidney transplant and dialysis patients [6–10]. An analysis of the REIN trial (mean estimated glomerular filtration rate [eGFR] 40 ml/min/1.73m^2^) also showed an increased incidence of ESRD in obese compared with non-obese patients [11]. Obesity is a major risk factor for the development of type 2 diabetes and hypertension, which are the primary causes of ESRD in US, Asia as well as in the Netherlands [1,9,12]. Other mechanisms by which obesity exerts its detrimental effects on kidney function decline are mediated via glomerular hyperfiltration, activation of the renin-angiotensin system, and the change of adipose tissue into a pro-inflammatory state that may cause focal and segmental glomerulosclerosis [13,14].

Obesity is a less important risk factor for death in the elderly compared with younger individuals in the general population [10]. We previously showed that in contrast to older incident dialysis patients, younger patients with obesity had a substantially elevated risk for death [9].

The aim of this study was to assess the extent to which obesity is a risk factor for start of dialysis, kidney function decline and mortality in patients on specialized pre-dialysis care. Since obesity is a potentially modifiable risk factor, it is important to identify patients who are susceptible to the detrimental effects of adiposity for targeted prevention of ESRD or premature death. Differences in the effect of obesity on ESRD and mortality between younger and older pre-dialysis patients have not been studied. Here we investigate the extent to which the effect of obesity on ESRD or death differs between younger (<65y) and older (≥65y) pre-dialysis patients. These results might inform care guidelines for pre-dialysis patients.

## Methods

### Study design and population

The PRE-dialysis PAtient REcord-2 (PREPARE-2) study is a prospective cohort study of incident pre-dialysis patients (≥18y) recruited in one of 25 nephrology specialized pre-dialysis outpatient clinics in the Netherlands, as previously described (S1 Appendix) [15]. Briefly, 502 pre-dialysis patients (eGFR <30 ml/min/1.73m^2^) with progressive loss of kidney function were included between July 2004 and June 2011, at the time of referral to a specialized pre-dialysis outpatient clinic. Patients with a failing kidney transplant who were transplanted at least one year ago were also eligible for inclusion. All patients were treated by their nephrologist in accordance with the treatment guidelines of the Dutch Federation of Nephrology, guidelines partly based on the K/DOQI and EBPG guidelines [16–18]. Patients were followed from the start of pre-dialysis care until start of dialysis, kidney transplantation, death or censoring. Censoring occurred in the event of refusal for further participation, recovery of kidney function, moving to an outpatient clinic not participating in the PREPARE-2 study, loss to follow-up or on October, 2016 (end of follow-up), whichever came first. This study was approved by the medical ethics committee or the institutional review boards (as appropriate) of all participating centers (S2 Appendix). All participants gave their written informed consent before study inclusion.

### Body mass index

To measure adiposity at baseline, we used body mass index (BMI), calculated as weight in kilograms divided by the square of height in meters. Following the World Health Organization (WHO) guidelines, obesity was defined as a BMI of 30 kg/m^2^ or greater, and overweight as a BMI of 25–29 kg/m^2^. We defined normal weight as a BMI of 20–24 kg/m^2^, which is within the normal range of 18–24 kg/m^2^ according to the WHO, and we considered this group *a priori* as the reference category [19]. We defined underweight as a BMI less than 20 kg/m^2^.

### Other demographic and clinical data

Data on demography, primary kidney disease, diabetes, history of cardiovascular disease (myocardial infarction and cerebrovascular accident), and medication prescriptions were collected at the start of specialized pre-dialysis care and at subsequent 6-month intervals. Patients were classified as either current smokers of cigarettes, former or never smokers. Laboratory data were extracted from the electronic hospital information systems or medical records. Primary kidney disease was classified according to the codes of the European Renal Association-European Dialysis and Transplantation Association [20]. We grouped patients into four classes of primary kidney disease: glomerulonephritis, diabetes mellitus, renal vascular disease, and other kidney diseases. We estimated GFR with the creatinine-based Chronic Kidney Disease Epidemiology Collaboration (CKD-EPI) equation from 2009 [21].

### Data analysis

Death and start of dialysis are competing risks. In older compared with younger pre-dialysis patients there is an increasing risk of death that precedes the onset of ESRD. Among younger pre-dialysis patients there were few deaths. Therefore, our study endpoint was a composite variable, ESRD that requires dialysis, or death. We censored follow-up at pre-emptive kidney transplantation. Since the effects of obesity may occur after a long induction period, we classified patients by their BMI at baseline [22]. We divided patients into two age categories, using 65y as the boundary point [9]. To compare the association of BMI and the combined endpoint between younger (<65y) and older (≥65y) pre-dialysis patients, patients were divided into eight categories based on their age (<65y or ≥65y) and baseline BMI: <20, 20–24, 25–29 and ≥30 kg/m^2^.

We assessed the relation between BMI categories and the composite endpoint using several methods. First, absolute incidence rates were calculated within each age and BMI category. Second, age-sex standardized dialysis-mortality rates were calculated for each BMI category, using 14 5-year age groups for each sex. Within each of the two broader age categories, age distribution differences across the four BMI categories were taken into account for men and women separately. Third, we calculated the age-sex standardized risk differences for outcome for the four BMI categories, taking normal BMI as the reference. Fourth, after stratification for age (<65y or ≥65y), we conducted a Cox proportional hazards (PH) regression analysis, obtaining hazard ratios (HR) estimating the effect of BMI and age on outcome, using a BMI of 20–24 kg/m^2^ of each group as the reference. Fifth, we performed the PH analyses within the two broad age groups (<65y or ≥65y) using a BMI of 20–24 kg/m^2^ in younger patients as the reference to allow for comparison of the effect of obesity between younger and older patients. For all PH analyses we present crude results, and also results adjusted for age, sex, and smoking. In a separate model we also adjusted for history of cardiovascular disease. We did not control for variables that we considered likely causal intermediates, such as hypertension, diabetes mellitus, and hypercholesterolemia [19]. In the PH regression models the proportionality assumption for each covariate was checked by adding the product term with the logarithm of follow-up time [23]. Finally, we evaluated the interaction between age (<65y or ≥65y) and obesity by assessing departures from additivity of effects [24–28]. We calculated the attributable proportion (AP) due to interaction, which is the proportion of the effect among patients with both risk factors that represents greater than additive effects.

Because start of dialysis, kidney transplantation and death are competing risks, we performed competing risk analyses for younger and older pre-dialysis separately. First, start of dialysis was taken as the event of interest, and death as the competing event. Second, start of dialysis or death was taken as the event of interest, and kidney transplantation as the competing event. According to the so-called subdistribution hazard (SH) approach, individuals who experience a competing event remain in the risk set instead of being censored, although they are no longer at risk of the event of interest [29]. The SH ratio (SHR) represents a ratio in a non-existing hypothetical population including those who experienced the competing event.

Finally, since starting dialysis is a shared clinical decision of the nephrologist based on prognosis, we also analyzed the effect of BMI on the rate of kidney function decline. We used a linear mixed model (LMM) to estimate the effect of BMI on the rate of decline in kidney function during the first two years of pre-dialysis care. All patients (n = 454) for whom kidney function was available at baseline were included in this analyses. The rationale for not using complete follow-up data is that stable patients who are still on pre-dialysis care after two years would contribute a relative large effect toward the average kidney function decline. The LMM takes into account that repeated measurements of the same patient are correlated [30].

For the standardization and calculation of risk differences we used Episheet (krothman.org/episheet.xls). For the CR and LMM analysis we used Stata version 14. This statistical software provides a method that fits competing risks regression models according to the SH method. All other aforementioned analyses were done using SPSS 24.0 (SPSS, Inc. Chicago, IL).

## Results

After excluding 10 patients, 5 with missing weight and 5 with missing height at baseline, 492 patients (98%) were included in the main analysis. The mean (±SD) age of the study cohort was 65±14 years, and 68% of the patients were men. The median (IQR) BMI was 26.0 (23.4–29.5) kg/m^2^, with an approximately normal distribution. The distribution of the pre-dialysis patients per category of BMI at baseline was: <20 (6%), 20 to 24 (36%), 25 to 29 (36%), and ≥30 (23%) kg/m^2^. Table 1 presents the baseline characteristics of the younger (<65 years) and older (≥ 65 years) pre-dialysis patients.

**Table 1:** Baseline characteristics of the cohort of incident pre-dialysis patients for younger (<65y) and older (≥65y) pre-dialysis patients.

Characteristics	Age <65y	Age ≥65y
Total No. (%)	212 (43)	280 (57)
Age, y	51.2 (10.2)	75.3 (5.7)
Men, No. (%)	134 (63)	199 (71)
Ethnicity, No. (%)		
white	187 (88.2)	267 (95.4)
black	5 (2.4)	11 (3.9)
asian	17 (8.0)	0 (0)
another	3 (1.4)	2 (0.7)
Primary kidney disease, No. (%)		
diabetes	28 (13)	40 (14)
glomerulonephritis	41 (19)	24 (9)
renal vascular disease	38 (18)	114 (41)
other	105 (50)	102 (36)
History of cardiovascular disease, No. (%)		
myocardial infarction	19 (9.0)	50 (17.9)
cerebrovascular accident	17 (8.0)	50 (17.9)
Smoking status, No. (%)		
current	78 (36.8)	152 (54.3)
former	47 (22.2)	51 (18.2)
never	87 (41.0)	77 (27.5)
Diabetes Mellitus, No. (%)	45 (21)	83 (30)
history of Diabetes mellitus, y	15 (5, 18)	12 (7, 20)
Weight (kg)	81.8 (18.4)	77.6 (15.2)
BMI (kg/m^2^)	27.2 (5.7)	26.5 (4.7)
<20 (low), No. (%)	13 (6.0)	16 (5.6)
20 to 24 (normal), No. (%)	77 (35.6)	98 (34.3)
25 to 29 (overweight), No. (%)	62 (28.7)	113 (39.5)
≥30 (obese), No. (%)	60 (27.8)	53 (18.5)
Waist (cm)		
men	103 (15.4)	103 (11.0)
women	95 (15.5)	98 (18.3)
Alcohol use (yes/no), No./Total No. (%)	104/212 (49.1)	125/278 (45.0)
Systolic blood pressure, mmHg	139 (20)	145 (23)
Diastolic blood pressure, mmHg	81 (11)	76 (12)
blood-pressure lowering drugs, No.(%)	175/187 (93.6)	229/240 (95.4)
eGFR ml/min per 1.73 m^2^	14.0 (10.6, 19.0)	14.5 (11.5, 18.1)
Total cholesterol (mmol/L)	4.6 (1.2)	4.3 (1.1)
statin use, No.(%)	103/187 (55)	150/240 (63)
CRP (mg/L)	4 (2–6)	6 (3–12)
Serum albumin (g/L)	41.7 (5.0)	40.0 (4.3)

Data are presented as median (interquartile range), mean (±SD) or number (percentage of the total).

BMI, body mass index; CRP, C-reactive protein; eGFR, estimated glomerular filtration rate.

The median (IQR) follow-up time for younger and older patients was 16 (7–31) and 16 (7–32) months, respectively. For younger patients the cumulative proportions that started dialysis or died by the end of follow-up was 66% and 4%, respectively, based on a life-table calculation, and for older patients these proportions were 64% and 14%. Table 2 shows the crude and age-sex standardized rates for the composite endpoint for younger and older pre-dialysis patients. S1 Table shows the crude rates for the separate endpoints of start of dialysis, mortality and kidney transplantation for younger and older pre-dialysis patients. The age-sex standardized composite endpoint rate was 2.3 times higher in younger obese patients than in younger patients with a normal BMI. In older pre-dialysis patients, differences in rates for the combined endpoint of start of dialysis or death were smaller between obese patients and those with a normal BMI. Thus, obesity appears to be a strong risk factor for start of dialysis or mortality among younger patients but a weaker risk factor among older pre-dialysis patients.

**Table 2:** Crude and age/sex standardized rates (95% CIs) and risk differences (95% CIs) for combined start of dialysis-mortality by BMI category for younger (<65 years) and older (≥65 years) pre-dialysis patients.

Age group	BMI (kg/m^2^)			
<65 years	<20	20 to 24	25 to 29	≥30
**Number of patients**	13	77	62	60
**Person years**	30.51	160.87	110.76	106.79
**Dialysis-deaths (total)**	7–0 (7)	43–1 (44)	44–4 (48)	48–2 (50)
**Dialysis-deaths/100 py**				
Crude	22.94	27.35	43.34	46.82
95% CI	11.40, 39.81	21.03, 34.68	34.41, 52.53	37.55, 56.13
Age-sex standardized	11.76	28.01	55.01	63.23
95% CI	2.03, 21.49	19.46, 36.57	3.27, 77.38	4.10, 85.43
**Risk difference**	-16.26	reference	27.00	35.21
95% CI	-29.21, -3.30	reference	3.06, 50.95	11.42, 59.01
**≥65 years**				
**Number of patients**	16	98	113	53
**Person years**	22.75	203.59	198.57	97.72
**Dialysis-deaths (total)**	12–3 (15)	52–19 (71)	74–13 (87)	42–3 (45)
**Dialysis-deaths/100 py**				
Crude	65.93	34.87	43.81	46.05
95% CI	44.89, 81.19	28.60, 41.57	37.01, 50.66	36.39, 55.57
Age-sex standardized	106.41	37.46	48.78	52.11
95% CI	12.70, 200.11	28.37, 46.54	34.58, 62.97	33.87, 70.35
**Risk difference**	68.95	reference	11.32	14.65
95% CI	-25.20, 163.10	reference	-5.54, 28.18	-5.73, 35.03

BMI: body mass index, CI: confidence interval, py: person year

We checked the PH assumption and no sign of violation was found. After stratification by age group (<65y or ≥65y) and adjustment for sex, smoking, and cardiovascular disease, we observed a U-shaped relation between BMI and start of dialysis-mortality among younger pre-dialysis patients, with a similar but milder pattern among older patients (Table 3). After multivariable adjustment the hazard ratios (95% CI) for the combined outcome for obesity were 1.81 for younger patients and 1.30 for older patients. In contrast to younger patients, in older pre-dialysis patients a low BMI at baseline was a stronger risk factor than obesity for the combined endpoint. Additional adjustment for serum albumin, baseline eGFR or alcohol consumption did not materially change the results (data not shown). Adjustment for diabetes, hypertension and dyslipidemia, attenuated the relation between obesity and outcome as expected. A sensitivity analysis in patients with diabetes showed a similar trend (data not shown). After exclusion of patients with a history of cardiovascular disease at baseline and adjustment for age, sex and smoking, the hazard ratios (95% CI) for the combined outcome by category of increasing BMI were for younger patients: 0.91 (0.39, 2.09), 1 (reference), 1.42 (0.89, 2,26) and 1.54 (0.95, 2.48), and for older patients: 1.62 (0.86, 3.04), 1 (reference) 1.22 (0.83, 1.81) and 1.21 (0.77, 1.90), respectively. After excluding patients with extremely low BMI (<18.5 kg/m^2^) the results did not materially change.

**Table 3:** Rate ratios of start of dialysis-mortality during follow-up, with 95% confidence intervals, from proportional hazards regression for BMI at baseline for younger (<65 years) and older (≥65 years) pre-dialysis patients. Normal BMI in each age category was taken as the reference category.

BMI per age group	Crude	Age and sex adjusted	Age, sex and smoking adjusted	Multivariable adjusted*
**Younger**				
**low BMI**	0.90 (0.40, 2.00)	0.90 (0.40, 2.02)	0.89 (0.40, 2.00)	0.92 (0.41, 2.09)
**normal BMI**	1.0 (reference)	1.0 (reference)	1.0 (reference)	1.0 (reference)
**high BMI**	1.63 (1.08, 2.45)	1.63 (1.08, 2.46)	1.66 (1.10, 2.50)	1.76 (1.16, 2.68)
**obese**	1.68 (1.12, 2.53)	1.70 (1.12, 2.59)	1.68 (1.10, 2.56)	1.81 (1.17, 2.81)
**Older**				
**low BMI**	1.86 (1.06, 3.26)	1.83 (1.04, 3.21)	1.77 (1.00, 3.13)	1.73 (0.97, 3.08)
**normal BMI**	1.0 (reference)	1.0 (reference)	1.0 (reference)	1.0 (reference)
**high BMI**	1.24 (0.91, 1.70)	1.24 (0.90, 1.70)	1.25 (0.91, 1.72)	1.25 (0.91, 1.71)
**obese**	1.31 (0.90, 1.90)	1.30 (0.89, 1.90)	1.31 (0.89, 1.91)	1.30 (0.89, 1.90)

* Multivariable adjustment: age, sex, smoking (current, former or never), co-morbidity (history of cardiovascular disease: myocardial infarction or CVA).

Our analysis to explore interaction between obesity and age (<65y or ≥ 65y) showed an AP due to interaction of -0.18, (Table 4). This result implies an effect of joint exposure that is less than the sum of the separate effects, a feature of biological antagonism. In contrast, an evaluation of the interaction between low BMI and age of ≥ 65y showed an AP of 0.48, indicating a joint effect of older age and low BMI that is considerably greater than the sum of their separate effects. Indeed, in Table 2, one notes that low BMI is a strong risk factor among the older patients but is associated with slightly lower risk among younger patients.

**Table 4:** Rate ratios of start of dialysis-mortality during follow-up, with 95% confidence intervals, from proportional hazards regression for BMI at baseline for younger (<65 years) and older (≥65 years) pre-dialysis patients. For interaction analysis normal BMI among younger patients was taken as the reference.

BMI per age group	Crude	Sex adjusted	Sex and smoking adjusted	Multivariable adjusted*
**Younger**				
**low BMI**	0.88 (0.40, 1.96)	0.88 (0.40, 1.97)	0.87 (0.39, 1.95)	0.88 (0.39, 1.97)
**normal BMI**	1.0 (reference)	1.0 (reference)	1.0 (reference)	1.0 (reference)
**high BMI**	1.61 (1.07, 2.42)	1.61 (1.07, 2.42)	1.63 (1.08, 2.45)	1.64 (1.09, 2.47)
**obese**	1.71 (1.14, 2.56)	1.71 (1.14, 2.56)	1.68 (1.12, 2.52)	1.69 (1.13, 2.55)
**Older**				
**low BMI**	2.32 (1.29, 4.17)	2.32 (1.29, 4.17)	2.16 (1.19, 3.91)	2.18 (1.20, 3.95)
**normal BMI**	1.28 (0.88, 1.86)	1.28 (0.88, 1.86)	1.26 (0.86, 1.83)	1.25 (0.86, 1.82)
**high BMI**	1.57 (1.09, 2.26)	1.57 (1.09, 2.26)	1.56 (1.08, 2.25)	1.55 (1.08, 2.24)
**obese**	1.65 (1.09, 2.50)	1.65 (1.09, 2.51)	1.64 (1.08, 2.48)	1.64 (1.08, 2.48)
**AP**				
**low BMI**	0.50 (0.32, 0.78)	0.50 (0.32, 0.78)	0.48 (0.29, 0.79)	0.48 (0.29, 0.79)
**obese**	-0.21 (-0.30, 0.06)	-0.21 (-0.30, 0.06)	-0.18 (-0.35, 0.09)	-0.18 (-0.36, 0.08)

*Multivariable adjustment: sex, smoking (current, former or never), co-morbidity (history of cardiovascular disease: myocardial infarction or CVA).

AP: Attributable Proportion to interaction with 95% CI. To quantify the amount of interaction on an additive scale, we calculated the AP = (HR++—HR_+-_—HR_-+_ +1)/HR_++_, which is the proportion of outcome that is due to interaction among patients with both exposures. Exposure refers to obese or low BMI versus normal BMI and old versus young pre-dialysis patients. In the absence of interaction on an additive scale AP equals 0.

If start of dialysis is taken as the event of interest and death is a censoring point, the HRs (95% CI) from the Cox-model after multivariable adjustment, for increasing category of BMI, were 0.92 (0.41–2.07), 1 (reference), 1.64 (1.06–2.52) and 1.77 (1.14–2.76) for younger patients and 1.81 (0.94–3.46), 1 (reference), 1.43 (1.00–2.05) and 1.64 (1.09–2.48) for older pre-dialysis patients. In the competing risk analysis, taking start of dialysis as the event of interest and death as the competing event, after multivariable adjustment the SHRs (95% CI) by category of increasing BMI were 0.90 (0.38–2.12), 1 (reference), 1.47 (0.96–2.24) and 1.72 (1.15–2.59) for younger patients and 1.68 (0.93–3.02), 1 (reference), 1.50 (1.05–2.14) and 1.80 (1.23–2.65) for older patients. The SHR of 1.72 in younger and 1.80 in older obese patients reflect the start of dialysis rate ratio among younger and older obese pre-dialysis patients who are alive or have already died without dialysis treatment. After taking start of dialysis or death as the event and kidney transplantation as the competing event, after multivariable adjustment the SHRs (95% CI) by category of increasing BMI were 1.17 (0.49–2.78), 1 (reference), 1.93 (1.27–2.93) and 1.96 (1.28–2.98) for younger patients and 1.88 (1.15–3.05), 1 (reference), 1.31 (0.95–1.80) and 1.42 (0.98–2.05) for older patients. Due to the low number of deaths in younger patients, we could not perform a competing risk analysis in which death could be taken as the event of interest (S1 Table).

**Fig 1:**
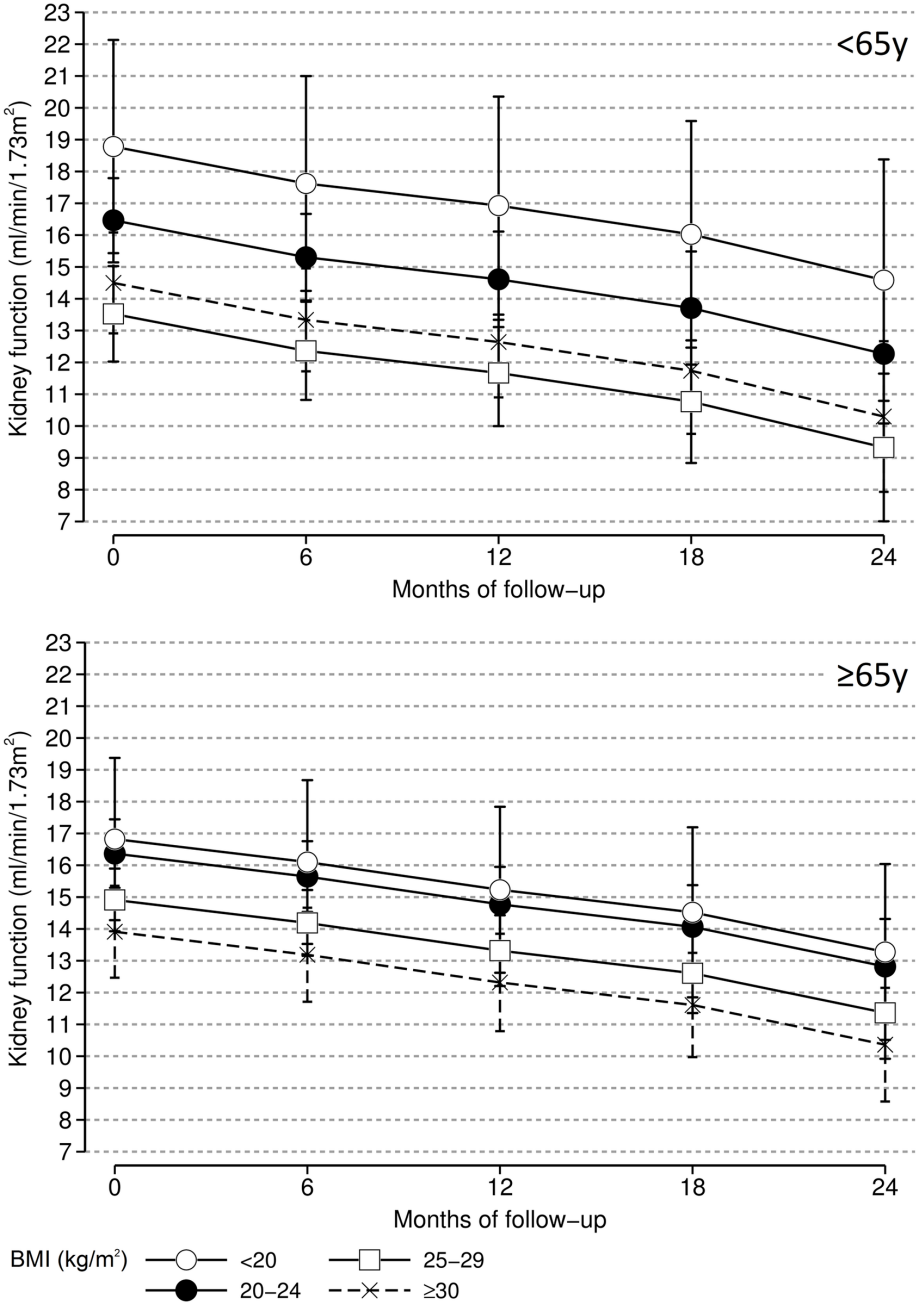
Kidney function decline in incident younger (<65 years) and older (≥65 years) pre-dialysis patients according to BMI category at baseline during 2 years of follow-up. Multivariable adjusted: age, sex, smoking (current, former or never), co-morbidity (history of cardiovascular disease: myocardial infarction or CVA).

eGFR measurements were available for 454 (94%) out of 482 pre-dialysis patients. The median number of eGFR measurements during the first 2 years of follow-up was 3 (IQR 2–4) and 10%, 33%, 57% of these patients had 1, 2 ≥3 measurements, respectively. The overall kidney function decline for all pre-dialysis patients was -0.15 (95% CI: -0.19, -0.12) ml/min/1.73m^2^ per month. There was no indication of effect modification between age and BMI with regard to eGFR decline. Fig 1 shows the multivariable adjusted kidney function decline according the four BMI categories at baseline during 2 years of follow-up for younger and older pre-dialysis patients separately. At baseline, after multivariable adjustment, the difference in kidney function (95%-CI) compared with the reference category was, for low BMI, 1.09 (-1.16, 3.35), and for high BMI -2.01 (-3.23, -0.79), and for obesity -2.06 (-3.45, -0.68) (S2 Table). Table 5 shows the crude and adjusted monthly kidney function change for the pre-dialysis patients according to the four BMI categories at baseline. Compared with patients with a normal BMI, after multivariable adjustment patients with obesity had similar renal function decline.

**Table 5:** Monthly kidney function decline with 95% confidence intervals among incident pre-dialysis patients during 2 years of follow-up according to BMI category at baseline.

BMI group	N	Crude	Age, sex and smoking adjusted	Multivariable adjusted*
**low BMI**	28	-0.12 (-0.36, 0.11)	-0.12 (-0.36, 0.11)	-0.15 (-0.38, 0.07)
**normal BMI**	163	-0.15 (-0.21, -0.09)	-0.15 (-0.21, -0.09)	-0.15 (-0.21, -0.09)
**high BMI**	163	-0.13 (-0.18, -0.08)	-0.13 (-0.18, -0.08)	-0.12 (-0.17, -0.07)
**obese**	100	-0.16 (-0.22, -0.10)	-0.16 (-0.22, -0.10)	-0.16 (-0.23, -0.10)

*Multivariable adjusted: age, sex, smoking (current, former or never), co-morbidity (history of cardiovascular disease: myocardial infarction or CVA).

## Discussion

This cohort study of incident pre-dialysis patients shows that obesity (BMI≥30 kg/m^2^), independent of important confounders, is associated with approximately 2-fold stronger risk for start of dialysis or death in younger (<65y) patients, compared with patients with normal weight. In older (≥65y) pre-dialysis patients, low BMI was a stronger risk factor than obesity for start of dialysis or mortality. Obese patients had a similar kidney function decline compared with patients with a normal BMI.

Our findings are consistent with large studies performed in individuals with early to moderate stages of CKD (stages 1–3) showing that a high BMI is a risk factor for ESRD [31–33]. A recent study (>3 million individuals, mean age 60y) showed that a BMI ≥30 kg/m^2^ is associated with rapid loss of kidney function (>5 ml/min/1.73m^2^ per year) in patients older than 40 years with eGFR>60 mL/min per 1.73 m^2^ [33]. A BMI between 25 and 30 kg/m^2^ was associated with lowest risk of kidney function decline. In contrast, a study in non-diabetic CKD stage 3–5 patients found little relation between obesity and RRT or kidney function decline [34]. That study, however, did not control for confounding by smoking, and patients with low BMI were grouped with the reference category, both of which may have contributed to an underestimation of the effect of obesity [35]. Another large cohort study showed little effect of obesity on risk of ESRD in patients with an eGFR <30 ml/min/1.73m^2^ [36]. However, smoking was likewise not controlled in that study, which again may have contributed to an underestimation of the effect of obesity. Furthermore, in contrast to our study with incident patients, the cohort comprised prevalent pre-dialysis patients, which may have resulted in survivor bias [37].

We took for our main analysis the composite outcome, start of dialysis or death, since death is a competing risk for start of dialysis, with risk of death increasing with age. In addition, starting dialysis is a shared decision of both the nephrologist and patient based on prognostic factors such as life expectancy and rate of kidney function decline, as well as uremic symptoms and fluid overload. For all age groups mortality rates increase substantially at eGFR<30 ml/min/1.73m^2^, being about 7% between 18-64y and 14% between 65-84y of age [38]. A 2-year follow-up study in CKD stage 4 patients reported 7% deaths and 25% RRT, and in an elderly population at 4 years 45% deaths and 23% RRT [10,13]. The relative risk for death associated with each level of kidney function decreases markedly by age as a result of higher background mortality risk [38]. A competing risk influences the opportunity for an event to occur. For example, in the present study a patient may have died before start of dialysis has occurred. We found in our competing-risk analysis a slightly higher risk of start of dialysis in obese patients compared with patients with a normal BMI. Younger obese pre-dialysis patients had a 1.7-fold increased risk and in older obese pre-dialysis patients we found a 1.8-fold increased risk for starting dialysis. In addition, older patients with a low BMI had about 1.7-fold increased risk of start of dialysis or death compared with those with a normal BMI, which might be attributable to malnutrition from identified or subclinical diseases [39].

In the PH analyses we censored at the time of kidney transplantation, which may have led to bias in estimating the effect of BMI as a result of differential selection for kidney transplantation, to the extent we could not control completely for covariates related to the selection for kidney transplantation. However, in the competing risk analyses, when taking kidney transplantation as the competing risk we found similar effects of obesity in younger and older pre-dialysis patients. In the Netherlands in 6 out of 7 kidney transplantation centers, pre-emptive kidney transplantation is suspended for patients with a BMI≥30 kg/m^2^, resulting in differential selection of healthy patients with a BMI<30 kg/m^2^ for pre-emptive kidney transplantation. In addition, pre-emptive kidney transplantation is scheduled for patients with an eGFR between 10–15 ml/min/1.73m^2^, whereas dialysis is commenced in patients with an eGFR <10 ml/min/1.73m^2^. Finally, the age of 70 years or older is a contra-indication for kidney transplantation if there is history of cardiovascular disease or diabetes.

The adverse effect of low BMI among older patients was notable, as was its absence among younger patients. The reason for this difference is unclear, as we have controlled for cardiovascular risk factors. It may simply be a chance finding. Both younger and older obese pre-dialysis patients had at baseline a lower kidney function compared with those with a normal BMI (Fig 1A and 1B). The lower kidney function may be a result of the history of obesity. The rate of kidney function decline was comparable among the four BMI categories during 24 months of follow-up. A longer follow-up might have shown larger differences in rate of function decline between the four BMI groups.

Our study had important limitations. First, risks related to obesity rise gradually, becoming appreciable only after at least 5 years of obesity [9]. In this study, we had no information about the duration of obesity. Second, we combined the outcomes, start of dialysis and death, because the number of deaths was too few to study the risks related to four BMI categories. Third, we used BMI as a proxy for obesity [40]. BMI does not fully reflect some age-related changes, such as the increasing proportion of body fat and decreasing muscle mass. Fourth, failure to control for unintentional weight loss could result in residual confounding. Loss of body mass, even in obese pre-dialysis patients, might result in greater mortality. We did not control for the amount of tobacco smoked by patients, which may have resulted in residual confounding. However, if anything, such residual confounding would tend to underestimate the effect of obesity. Finally, our results may not apply to all CKD 4–5 patients, as we studied only those receiving specialized pre-dialysis care in an outpatient clinic.

In conclusion, younger obese pre-dialysis patients have an increased risk for start of dialysis and death compared with those with a normal BMI. In contrast, underweight was a stronger risk factor than obesity in older pre-dialysis patients for start of dialysis and death.

## Supporting information

S1 Appendix Protocol PREdialysis PAtient Records (PREPARE) study. (PDF)

S2 Appendix Ethics statement. (PDF)

S1 Table Crude incidence rates for mortality, dialysis, and kidney transplantation (95% CIs) by BMI category for younger (<65y) and older (≥65y) pre-dialysis patients. BMI, body mass index; NTx, kidney transplantation.
(PDF)

S2 Table Baseline difference of kidney function (95% CI) according to BMI category for incident pre-dialysis patients compared with the reference category with normal BMI. Multivariable adjusted: age, sex, smoking (current, former or never), co-morbidity (history of cardiovascular disease: myocardial infarction or CVA). BMI, body mass index.
(PDF)
