# Supplementary material for: Obesity and risk of death or dialysis in younger and older patients on specialized pre-dialysis care
Source: PLoS One. 2017 Sep 5;12(9):e0184007. doi: 10.1371/journal.pone.0184007 (PMC5584800; doi:10.1371/journal.pone.0184007)
Supplement: S1 Appendix — (PDF) [file pone.0184007.s001.pdf]

PREPARE

PREdialysis PAtient REcords study

protocolnr.: P04.136

Yvo Sijpkens

Jeannette van Manen

Friedo Dekker

Departments of Kidney Diseases and Clinical Epidemiology, LUMC

March 2005

Version 6

1. PREPARE

1a. PREPARE = PREdialysis PAtient REcord study

PREPARE is a prospective Dutch cohort study in patients with severe chronic renal insufficiency (renal clearance <30 ml/min), who are prepared for renal replacement therapy, in whom the influence of different risk factors on decline of kidney function, cardiovascular disease, and survival are studied. This prospective study is associated with the ongoing retrospective PREPARE study in pre-dialysis patients.

1b. PREPARE study group:

Yvo Sijpkens, internist-nephrologist
Department of Kidney Diseases
LUMC, post zone C3P, P.O. Box 9600, 2300 RC Leiden
tel: 071-5262209/2148, mail: y.w.j.sijpkens@lumc.nl

Jeannette van Manen, epidemiologist
Department of Clinical Epidemiology
LUMC, post zone C9P, P.O. Box 9600, 2300 RC Leiden
tel: 071-5261384, mail: j.g.van_manen@lumc.nl

Roel Huisman, internist-nephrologist
Dialysis Center Groningen
P.O. Box 30.001
9700 RB Groningen
tel: 050-3616161, mail: r.m.huisman@int.azg.nl

Els Boeschoten, internist-nephrologist
Hans Mak Institute
Koningin Wilhelminalaan 29b
1411 EL Naarden
tel: 035-6783000, mail: e.boeschoten@hansmakinstituut.nl

Friedo Dekker, clinical epidemiologist
Department of Clinical Epidemiology
LUMC, post zone C9P, P.O. Box 9600, 2300 RC Leiden
tel: 071-5262183 \ 5230, mail: f.w.dekker@lumc.nl

1c. Sponsor:

PREPARE is financially supported by an unrestricted grant from Amgen BV.

1d. Version protocol

6

1e. Date protocol:

March 2005

2. INTRODUCTION

2a. Background and rationale

The incidence and prevalence of chronic renal insufficiency in Europe are rapidly increasing (1). Due to obesity and an ageing population, diabetic nephropathy and nephrosclerosis are currently the main causes of renal failure. In order to improve prognosis of kidney patients in 2002 K/DOQI guidelines for chronic kidney insufficiency (CKI) have been published (2). The NfN (Dutch Nephrology federation) guideline for internists has been based on this (3). Based on kidney damage and renal clearance, a categorization has been developed, in which different stages are linked to an action plan. In patients with CKI with a renal clearance of less than 30 ml/min (stage IV) this includes the inhibition of decline of kidney function, the prevention and treatment of metabolic complications, including cardiovascular diseases, and the preparation for renal replacement therapy.

Patients with severe CKI are prone to further decline of kidney function and the need of renal replacement therapy. The decline of kidney function depends on the underlying cause and the presence of progression factors such as hypertension, proteinuria, and smoking (4). With strict regimens and the strongly improved pharmacotherapy, even in this stage, it is possible to oppose or attenuate decline of kidney function (5). For an optimal estimation of time to renal replacement therapy in the individual patient however, more detailed knowledge of the determining factors is needed.

In patients with CKD stage IV metabolic complications, anemia, secondary hyperparathyroidism, and acidosis are highly prevalent. Due to the presence of traditional and renal determined risk factors also a strongly increased risk of cardiovascular diseases is present (6). This influences the increased mortality of patients with chronic kidney disease, patients on dialysis, and transplanted patients (7, 8). To improve the prognosis of patients with chronic kidney disease it is opportune to start in an early phase with optimal prevention of cardiovascular diseases. To this end, a better insight in the impact of cardiovascular risk factors and treatment on morbidity and mortality is needed.

In CKD stage IV an eligible patient will be prepared for dialysis and transplantation. Because of donor shortage the number of transplantations with living kidney donors has been strongly increased. This provides the opportunity to transplant preemptively and prevent the negative consequences of severe chronic kidney insufficiency and dialysis (9). In view of the long waiting list for kidney transplantation the patients who need a transplant with a post mortal donor will benefit from this development.

Timely referral to an internist-nephrologist is needed for well performance of the action plan and likewise to improve the prognosis of the patient (10). For implementation of the preventive measures more intensive care and patient education is needed (11). This requires multidisciplinary patient care with support of specialized nurses, dieticians, and social workers. To this end, more and more specialized outpatient clinics have been developed for patients with severe CKI. It can be expected that the quality of this care process will become part of the certification procedure of dialysis centers. For the establishment and planning of these specialized outpatient clinics, more data are needed on the size of the patient population and the expected decline of renal function.

2b. Previous research

In 2002 the retrospective part of PREPARE has been started with the primary research question to investigate which factors influence time to renal replacement therapy. Patients who were registered in the period 1999-2001 as new pre-dialysis patients were included. In general this was the time of start with preparations for dialysis treatment. Research nurses from the Hans Mak Institute performed research in medical files during which characteristics of these patients were collected at start of pre-dialysis care and at end of follow-up (start renal replacement therapy, death, end of follow-up). In order to estimate the decline of kidney function all creatinine estimates derived from the hospital information system were recalculated to creatinine clearance by the Cockcroft formula.

In a first analysis the parameters at inclusion were associated with time to renal replacement therapy or end of follow-up. Incident (1999-2001) pre-dialysis patients from the LUMC, AMC, and AZG were followed till time of start of renal replacement therapy, death or January 1, 2003. In addition to poor kidney function, young age, anemia, diabetes, and cystic kidney disease were indicators for a faster need for renal replacement therapy. A second analysis showed that young age, male gender, cystic kidneys, and proteinuria are indicators for a faster decline of kidney function. Enlargement of this study with several peripheral centers with a reliable registration of pre-dialysis patients resulted in a group of more than 500 retrospective patients enabling reliable analyses on renal and cardiovascular endpoints. Final results are to be expected soon.

The inherent limitations of this retrospective study are that only data that were available in the patient records were used. Important parameters are not always complete or were recorded with irregularities. Blood or urine and DNA samples were not collected. Data on patients' quality of life are lacking. During these years, the nationwide guidelines for patients with CKD were not used.

3. WHAT IS THE AIM OF PREPARE?

3a. Objective

The first objective of PREPARE is to assess factors which influence the degree of decline of kidney function in patients with severe CKI and likewise the time to renal replacement therapy. Based on the results it should be easier to estimate whether and when a patient has to start with preparations for dialysis and transplantation. The second objective is to determine traditional and renal determined risk factors for the prediction of cardiovascular disease in patients with severe CKI. Based on this knowledge doctors should be able to better prevent disease burden and premature death. This study provides a good description of the pre-dialysis population, including data on quality of life.

PREPARE aims to improve the insight into the pathophysiological mechanisms of decline of renal function and cardiovascular burden in order to improve the associated interventions. PREPARE should stimulate early referral to pre-dialysis care, thereby leading to, in addition to multidisciplinary care, good preparation for dialysis. Furthermore, pre-emptive transplantation with a living donor will become more feasible for many patients.

3b. Research questions

1. Which factors determine the decline of kidney function and the time to renal replacement therapy in patients with severe CKI?

2. What are the risk factors for cardiovascular morbidity and mortality in patients with severe CKI?

3. What is the quality of life in patients with severe CKI and which factors are influencing this?

4. EXECUTION

4a. Selection of patients

In principle every dialysis center in The Netherlands can participate in the study. For practical reasons the data collection can best be performed in a present or to be created pre-dialysis outpatient clinic. Incident pre-dialysis patients who fulfill the following inclusion criteria can participate in the study:

- 18 years or older
- informed consent
- under treatment by internist-nephrologist
- chronic kidney insufficiency stage IV (Cockcroft clearance <30 ml/min)
- in case of chronic transplant failure at least one year after transplantation
- eligible for renal replacement therapy
- less than six months under treatment at the ‘pre-dialysis outpatient clinic’

4b. Data collection

PREPARE uses data that will, according to the current NfN guideline, be collected routinely in a pre-dialysis outpatient clinic. Some additional demographical data will be collected together with a quality of life questionnaire, and biological material (serum, urine, and a one-off full-blood sample) for future analyses. If possible, vascular examination such as IMT (intima media thickness) or PWV (pulse wave velocity) measures will be performed. All data will be collected in a CRF at study inclusion and subsequently every six months thereafter until start of dialysis or transplantation (end of study). This can be mainly performed by a nurse practitioner, nurse or dietician. Lab results will be collected retrospectively from the hospital information system (HIS). Details of the data to be collected can be found in Appendix I.

After the initiation of renal replacement therapy nationwide dialysis and transplantation registries will be used to collect data on the occurrence of cardiovascular morbidity and mortality.

4c. Treatment of patients

For the treatment of patients participating in PREPARE the recent NfN guidelines for patients with CKI are applicable (3). In this way, treatment is closely related to the current practice in pre-dialysis outpatient clinics in which the multidisciplinary approach by nephrologist, nurse, dietician, and social worker is frequently used. Every patient who is eligible for renal replacement therapy will, according to current practice, be informed about dialysis and transplantation. Also the possibilities of pre-

emptive transplantation with a living donor, possibly by means of a cross-over procedure, will come up for discussion.

5. DURATION AND SCOPE OF PREPARE

5a. Number of patients

PREPARE aims to include at least ten centers to participate with a yearly contribution of 30-50 patients so finally at least 500 patients will participate. Based on preliminary analyses of the retrospective part of PREPARE it appears that this number of patients is needed to answer research questions and to distinguish relevant subgroups, such as diabetes and status after transplantation. Furthermore, the retrospective analyses showed that the seven centers under study in the period 1999-2001 had a mean of 38 new patients per year, with an increase in the number of new patients over time because of earlier inclusion in the pre-dialysis phase.

5b. Time plan

The present protocol has already been approved by the medical ethical committee of the LUMC. The official start date will be October 1, 2004. The inclusion period will last, depending on the number of patients included, till January 1, 2006, with a follow-up of at least twelve months per patient.

5c. Logistical procedures

The execution of the study will be coordinated by each individual center and embedded in routine care thereby restricting the additional time spent on this protocol to the minimum. A CRF is available for inclusion, follow-up, and exclusion parameters, which will be filled out by doctor and nurse. These forms will be checked and collected by employees from the Hans Mak Institute and will be scanned and transferred to a database. Cellular material for DNA analysis and blood and urine samples, justified locally in two serum and urine tubes, will be collected periodically and stored in fridges at the LUMC.

6. STATISTICAL ANALYSIS

6a. Statistical methods

Start of renal replacement therapy and the degree of loss of kidney function will be used as renal endpoints. The degree of loss of kidney function will be estimated by calculating the decline of creatinine clearance per month based on the data collected from the LABZIS. The influence of possible risk factors will be studied by linear regression and repeated measures analysis of variance. The time between inclusion and the start of renal replacement therapy will be analyzed by Kaplan-Meier and Cox regression analysis adjusted for instance for kidney function at inclusion. Relevant subgroups (such as primary kidney disease, early versus late referral, previous renal replacement therapy etc.) will be analyzed separately. All-cause mortality, cardiovascular mortality, and the occurrence of cardiovascular morbidity (acute coronary syndrome, objectified peripheral vascular disease, CVA) are the other endpoints that will be analyzed by Cox regression. The influence of clinical parameters on the patients' perceived quality of life will be analyzed with linear and logistic regression analysis.

The change in quality of life during time will be studied by repeated measures analysis.

6b. Execution of statistical analysis

Analysis and reporting will be performed by junior researches (promovendi) supervised by members of the PREPARE study group.

7. ADMINISTRATIVE PROCEDURES

7a. Coding of personal data

The data from the included patients will be coded locally with a subsequent number within a center. The list with names and patient numbers remains in the centers under management of the nephrologists. Names and identification numbers will not be transferred to the researchers. The nephrologist in turn has no access to the individual study results.

7b. Data collection

The data collection will be coordinated by the Hans Mak Institute, which also coordinates the data collection of other multicenter studies (among others NECOSAD, a large multicenter cohort study among about 2000 new dialysis patients). Data will be collected locally by a trained nurse and/or dietician under responsibility of the nephrologist. Lab results will be collected from the LABZIS. A few times a year a trial nurse from the Hans Mak Institute will monitor the coded data and collect them for further processing.

8. ADDITIONAL INFORMATION – DNA EXAMINATION

All patients will once provide a full blood sample -at inclusion- for DNA extraction. DNA will be collected to determine polymorphisms that are relevant for the cause and consequences of renal failure. This will especially be polymorphisms which are of importance for the development and course of cardiovascular morbidity, since these play an important role in renal failure.

Coding/anonymisation of the material will be performed, since the DNA data have to be linked to other phenotypical data. The researchers will not be able to link the data to individual persons. The key of this coding will be kept by the treating nephrologist, who in turn has no access to the individual study results. Blood and DNA samples will only be traceable to patient level with the aid of the treating nephrologist. Results from the DNA examination will not be announced to the patient.

Blood draw takes place within the center. The material will be coded and subsequently transported to the LUMC where DNA isolation takes place. Remaining blood and DNA will be stored in the LUMC under responsibility of the PREPARE study group.

The blood and DNA will -even after death of the patient- during the course of the study be stored to enable genetic research. The study ends when the last paper has been written. The material will be stored till ten years after the end of the study, thereafter the material will be destructed.

9. CONFLICT OF INTEREST

The sponsor had no influence on the creation of the protocol and will not be involved in the data analysis. Publications will be written by the PREPARE study group. Manuscripts will be given to the sponsor for inspection during 30 days. Decisions on the content of manuscripts will be made by the PREPARE study group without any influence of the sponsor.

10. INFORMED CONSENT

Every patient who fulfills the inclusion criteria will be informed by patient information form (Appendix II) about the aim, nature, and duration of the study. Consent will be asked for the collection of cell- and stored material. Before study inclusion a written consent will be asked, which will also be filled out by the nephrologist.

11. REFERENCES

1. Stengel B, Billon S, van Dijk PCW et al. Trends in the incidence of renal replacement therapy for end-stage renal disease in Europe, 1990-1999. Nephrol Dial Transplant 2003:18: 1824-1833.
2. K/DOQI clinical practice guidelines for chronic kidney disease: evaluation, classification, and stratification. Kidney Disease Outcome Quality Initiative. Am J Kidney Dis 2002:39: S1-246.
3. Ter Wee PM, Jorna ATM. Behandeling van patiënten met chronische nierinsufficiëntie; richtlijn voor internisten. Ned Tijdschr Geneeskd 2004:148: 719-724.
4. Jafar TH, Stark PC, Schmid CH et al. Progression of Chronic Kidney Disease: The Role of Blood Pressure Control, Proteinuria, and Angiotensin-Converting Enzyme Inhibition: A Patient-Level Meta-Analysis. Ann Intern Med 2003:139: 244-252.
5. Ruggenenti P, Perna A, Remuzzi G. ACE inhibitors to prevent end-stage renal disease: when to start and why possibly never to stop: a post hoc analysis of the REIN trial results. Ramipril Efficacy in Nephropathy. J Am Soc Nephrol 2001:12: 2832-2837.
6. Foley RN, Parfrey PS, Sarnak MJ. Epidemiology of cardiovascular disease in chronic renal disease. J Am Soc Nephrol 1998:9: S16-S23.
7. Merkus MP, Jager KJ, Dekker FW, de Haan RJ, Boeschoten EW, Krediet RT. Predictors of poor outcome in chronic dialysis patients: The Netherlands Cooperative Study on the Adequacy of Dialysis. The NECOSAD Study Group. Am J Kidney Dis 2000:35: 69-79.
8. Arend SM, Mallat MJ, Westendorp RJ, van der Woude FJ, van Es LA. Patient survival after renal transplantation; more than 25 years follow-up. Nephrol Dial Transplant 1997:12: 1672-1679.
9. Kasiske BL, Snyder JJ, Matas AJ, Ellison MD, Gill JS, Kausz AT. Preemptive kidney transplantation: the advantage and the advantaged. J Am Soc Nephrol 2002:13: 1358-1364.
10. Stack A. Impact of timing of nephrology referral and pre-ESRD care on mortality risk among new ESRD patients in the United States. American Journal of Kidney Diseases 2003:41: 310-318.
11. St Peter W, Schoolwerth A, McGowan T, McClellan W. Chronic kidney disease: Issues and establishing programs and clinics for improved patient outcomes. American Journal of Kidney Diseases 2003:41: 903-924.

Appendix I: PREPARE parameters

The NfN guideline for chronic kidney insufficiency (CKI) states the following with regard to routinely based data collection:

Patients with CKI stage three onwards (creatinine clearance 30-60 ml/min) have to be checked at least two to four times a year, whereby blood pressure and weight will be measured as well as hemoglobin, hematocrit, urea, creatinine, potassium, calcium, phosphorus, albumin, PTH, bicarbonate, and lipids in the laboratory. The course of kidney function can be followed by the reciprocal value of serum creatinine; the creatinine clearance (measured with 24-hr urine collection or estimated using the Cockroft-Gault or MDRD formula). Proteinuria can best be measured in 24-hr urine or as protein (albumin)/creatinine ratio in a urine sample.
More generally within the NfN guideline the importance of swift recognition of cardiovascular risk factors and complications has been indicated. For that reason in all patients at inclusion in PREPARE an ECG and echo will be made (to estimate left ventricular hypertrophy), which in many centers is routinely practice within the framework of the preparation for kidney transplantation.
Below, by method of data collection, the specific data to be collected are listed:

Recording form (*at inclusion /every six months /***optional):**
Anamnesis
*Demographic data (age, sex, race)
*Primary kidney disease (EDTA code, date of diagnosis, and if present biopsy)
*Comorbidity (hypertension, diabetes, CVA, peripheral and coronary vascular disease, heart failure)
(new events will be mentioned on the follow-up forms)
**Intoxications (smoking, alcohol)
**Medication (class, type, and dose)
**Questionnaire Quality of life KD-QOL (= SF-36 completed with disease specific part for people with kidney diseases)

Physical examination
**blood pressure
*24-hr blood pressure
**pulse
*length
**weight (body mass index)
**abdominal circumference

Laboratory
***Blood*
hemoglobin
hematocrit
urea
creatinine (-> Cockcroft clearance and MDRD clearance)
potassium
bicarbonate
calcium
phosphorus
albumin
alkaline phosphatase
parathormone
25 (OH) vit D

1,25 (OH)2 vit D
cholesterol
LDL-cholesterol
HDL-cholesterol
triglycerides sober
glucose sober
insulin sober
HbA1c
CRP
fibrinogen
ferritin
iron saturation
*homocystein
*folic acid
*vitamin B12
stored serum (2 x 5 ml)
*cells for DNA

***Urine sample*
sediment
total protein/creatinine ratio
albumin/creatinine ratio
osmolality

***24-hr urine*
urea (-> protein intake = urea x 0.18 + 15)
creatinine (-> endogenous creatinine clearance)
total protein
albumin
sodium
calcium
phosphorus
stored urine (2 x 5 ml)

Additional examinations
*ECG
*echo
***echo carotis (IMT)
***pulse wave velocity

Appendix II: Patient information form

Patient information for a scientific research project on the factors that influence the decline of kidney function and the development of cardiovascular diseases in patients who are prepared for renal replacement therapy.

Dear Sir/Madame,

With reference to the conversation with your treating physician you receive the written information concerning a scientific research project – the PREPARE study – for which your cooperation has been asked.

Background
PREPARE is a Dutch study in patients with chronic kidney failure who are being prepared for renal replacement therapy (dialysis or transplantation). In this research it will be studied whether it is possible to better estimate time to renal replacement therapy as compared to the current situation. It is of importance for patients to know where they stand, but it is also important for the physicians to know, because then they know when to start with which preparations. Furthermore the study aims to find which factors influence the development of cardiovascular diseases. Cardiovascular diseases are relatively frequent in patient with chronic kidney failure. To improve the prognosis of kidney patients, also during dialysis and after transplantation, it is of importance to treat these cardiovascular diseases in an early stage as possible. By studying factors which influence the development of cardiovascular diseases a better insight can be obtained in the possibilities for their prevention.

Aim of the study
So, the aim of the study we are asking your cooperation for is (1) to better estimate the time which is needed to renal replacement therapy and (2) to get more insight in the factors which influence the development and prevention of cardiovascular diseases.

Study design
All people with chronic renal failure aged 18 years and over who are being prepared for renal replacement therapy are eligible for the study. The study involves the monitoring of disease progression with the regular treatment. Since the regular treatment will be continued, this means that you can visit your nephrologist for a check-up at the outpatient clinic with your regular scheme every 4-8 weeks. As usual during your outpatient clinic visit your medication can be adjusted based on your (possible) complaints, together with the laboratory results from blood and urine. Since you are indicated to possibly receive renal replacement therapy, your nephrologist might ask for additional medical examinations to better monitor your health status. This is however, part of regular treatment. Additional examinations might include for example ECG or echo of the hearth to

study the heart function or an echo of the carotis to better monitor your blood vessels. Not every patient will get additional examinations, these will only be performed on indication.

Data which are collected during your regular monitor visits at the outpatient clinic will be used as much as possible. For example blood and 24-hr urine measurements will also be used for the scientific research to look whether specific substances in blood and urine are associated with the development of cardiovascular diseases. Your medical data will be filled out on a form about your health status by your physician. Our additional question to you is to fill out a questionnaire about how you are feeling. This questionnaire will be filled out at inclusion and subsequently every six months. Furthermore, at inclusion and subsequently every six months your nutritional status will be monitored. This happens by means of a number of questions concerning your weight and dietary habits, and a short physical examination by a nurse. Finally, at inclusion a one-off additional blood tube will be collected. By means of the DNA of these blood cells it can be studied which role hereditary factors play in the decline of kidney function and the development of cardiovascular diseases.

Burden of participants

The study will take at least one year until start date of renal replacement therapy. Blood draw, the collection of urine, and the medical data will be collected even without the study, so this means no additional burden. Within the study period you will be asked two times a year to fill out a questionnaire on the quality of life. We estimate it will take you about one hour each time. No risks are present. Personal results of the study -including DNA measurements- will be communicated with nobody.

Voluntarily of participation

Your participation in this study is voluntary. If you agree to participate in this study, you have always the liberty to withdraw your decision. You do not have to give a reason for this. Whether you will participate or not will not influence your further treatment or your understanding with your physician.
Your treating physician can also stop your participation in this study if he or she thinks it is in your best interest. He or she will discuss this with you. Please, take your time before you will make your decision to participate or not. Then you can discuss this information with your partner, family, general practitioner, or others.

Confidentiality of data

All your data which will be collected during the research will be treated with confidentiality and no unauthorized persons will have insight in your data. In addition, your data will be anonimyzed so the researchers will not be able to link your data with your name. For that reason only a study code and no name will be used on the forms with the research data. Also urine, blood, and DNA will be supplied with a code and not your name. Only your physician will know who is linked to which research code.

Finally

If you have further questions concerning this study you can contact an independent physician of the LUMC, Dr. P. van der Boog, nephrologist (tel.: 071-5262148).

Researcher: Dr. Y.W.J. Sijpkens
Function: internist-nephrologist
Department of Kidney Diseases, LUMC

Informed consent for participation in PREPARE

Name patient: ____

Date of birth: ____

Sex: male/female

Center: ____

I, undersigned Madame/Sir ...., declare that Dr. .... invited me to participate in the PREPARE study

I declare:

1. that the content of PREPARE was explained to me.
2. that I am insured that I will receive the treatment which is regular in this hospital.
3. that I am asked at inclusion and subsequently every six months for at least 1 year to fill out a questionnaire.
4. that I am insured that all data transfer within the PREPARE study will be anonimyzed.
5. that my blood and urine can be used for the determination of risk factors in patients with chronic renal failure.
6. that I have read the patient information and that all my questions have been answered.

I hereby declare that I am willing to participate in the PREPARE study for which I give my authorization to make available both the data of my treatment as well as the data from the questionnaires, and the laboratory results in anonymized form for the PREPARE study.

Signature patient Signature treating physician

Date

Study number: .... (to be filled out by the nephrologist)
