# Supplementary material for: Obesity and risk of death or dialysis in younger and older patients on specialized pre-dialysis care
Source: PLoS One. 2017 Sep 5;12(9):e0184007. doi: 10.1371/journal.pone.0184007 (PMC5584800; doi:10.1371/journal.pone.0184007)
Supplement: S1 Table — BMI, body mass index; NTx, kidney transplantation. (PDF) [file pone.0184007.s003.pdf]

	BMI (kg/m^2)			
**<65 years**	**<20**	**20 to 24**	**25 to 29**	**≥30**
**Number of patients**	13	77	62	60
**Person years**	30.51	160.87	110.76	106.79
**Deaths**	0	1	4	2
**Deaths/100 person-years**	0	0.62	3.60	1.87
95% CI	NA	0.11, 3.43	1.41, 8.90	0.51, 6.56

	BMI (kg/m^2)			
**≥65 years**	**<20**	**20 to 24**	**25 to 29**	**≥30**
**Number of patients**	16	98	113	53
**Person years**	22.75	203.59	198.57	97.72
**Deaths**	3	19	13	3
**Deaths/100 person-years**	13.19	9.33	6.55	3.07
95% CI	4.54, 32.13	6.04, 14.09	3.86, 10.85	1.05, 8.62

	BMI (kg/m^2)			
**<65 years**	**<20**	**20 to 24**	**25 to 29**	**≥30**
**Number of patients**	13	77	62	60
**Person years**	30.51	160.87	110.76	106.79
**Start of dialysis**	7	43	44	48
**Dialysis/100 person-years**	22.94	26.73	35.72	44.95
95% CI	11.40, 39.81	20.48, 34.03	31.03, 48.93	35.78, 54.30

	BMI (kg/m^2)			
**≥65 years**	**<20**	**20 to 24**	**25 to 29**	**≥30**
**Number of patients**	16	98	113	53
**Person years**	22.75	203.59	198.57	97.72
**Start of dialysis**	12	52	74	42
**Dialysis/100 person-years**	52.75	25.54	37.27	42.98
95% CI	32.96, 70.76	20.00, 31.89	30.77, 44.08	33.51, 52.74

	BMI (kg/m^2)			
**<65 years**	**<20**	**20 to 24**	**25 to 29**	**≥30**
**Number of patients**	13	77	62	60
**Person years**	30.51	160.87	110.76	106.79
**Kidney transplantation**	3	23	9	7
**NTx/100 person-years**	9.83	14.30	9.97	6.55
95% CI	3.35, 24.90	9.71, 20.52	4.32, 14.69	3.20, 12.89

	BMI (kg/m^2)			
**≥65 years**	**<20**	**20 to 24**	**25 to 29**	**≥30**
**Number of patients**	16	98	113	53
**Person years**	22.75	203.59	198.57	97.72
**Kidney transplantation**	0	5	1	0
**NTx/100 person-years**	0	2.46	0.50	0
95% CI	NA	1.05, 5.61	0.09, 2.79	NA
