# Supplementary material for: Obesity and risk of death or dialysis in younger and older patients on specialized pre-dialysis care
Source: PLoS One. 2017 Sep 5;12(9):e0184007. doi: 10.1371/journal.pone.0184007 (PMC5584800; doi:10.1371/journal.pone.0184007)
Supplement: S2 Table — Multivariable adjusted: age, sex, smoking (current, former or never), co-morbidity (history of cardiovascular disease: myocardial infarction or CVA). BMI, body mass index. (PDF) [file pone.0184007.s004.pdf]

	N	Crude	Age and sex adjusted	Age, sex and smoking adjusted	Mutivariable adjusted
**low BMI**	28	0.74 (-1.50, 2.99)	0.98 (-1.27, 3.23)	0.93 (-1.33, 3.19)	1.09 (-1.16, 3.35)
**normal BMI**	163	1 (reference)	1 (reference)	1 (reference)	1 (reference)
**high BMI**	163	-1.88 (-3.10, -0.67)	-2.03 (-3.25, -0.81)	-2.02 (-3.24, -0.79)	-2.01 (-3.23, -0.79)
**obese**	100	-2.20 (-3.59, -0.81)	-2.20 (-3.59, -0.81)	-2.16 (-3.55, -0.77)	-2.06 (-3.45, -0.68)
